# Electroacupuncture in the treatment of gastrointestinal dysfunction after laparoscopic nephrectomy: a retrospective analysis

**DOI:** 10.1186/s12906-025-04894-y

**Published:** 2025-04-25

**Authors:** Yang Zhenjie, Zhang Xiang

**Affiliations:** 1 Department of Acupuncture - Moxibustion and Tuina, Qilu Hospital of Shandong University, Jinan, 250012 China; 2 Department of Urology, Qilu Hospital of Shandong University, No. 107 Wenhuaxi Road, Lixia District, Jinan, 250012 China

**Keywords:** Acupuncture, Laparoscopic nephrectomy, Postoperative Gastrointestinal dysfunction (POGD), Efficacy evaluation, Retrospective analysis

## Abstract

**Background:**

Although the Enhanced Recovery After Surgery (ERAS) protocol has been optimised, postoperative gastrointestinal dysfunction (POGD) still significantly hampers recovery after laparoscopic nephrectomy, thus adding to the burden on the national healthcare system. We aimed to evaluate the efficiency of electroacupuncture (EA) in reducing the duration of POGD, while exploring the pertinent factors.

**Methods:**

A total of 226 medical records of patients with POGD from January 1 to October 31, 2022, were collected and analyzed. They were administered the ERAS protocol for urology and divided into Early Acupuncture Group (Observation Group 1), Delayed Acupuncture Group (Observation Group 2), and Non-Acupuncture Group (Control Group). The primary outcome was the duration of the first anal exhaust and defecation after surgery. And the visual analogue scale (VAS) score of abdominal distension, shoulder pain, nausea, and adverse events within 72 h after surgery were observed.

**Results:**

Cases of the three groups were 79, 71, and 76. The anal exhaust time was 40.59 ± 18.21, 54.54 ± 12.88, and 62.26 ± 15.79 h, while the first defecation time was 56.28 ± 12.21, 71.13 ± 11.29, and 78.36 ± 14.71 h respectively. The operation duration affected the anal exhaust time (*P* < 0.05). Acupuncture timing affected recovery time statistically (*P* < 0.05). The VAS for abdominal distension, shoulder pain and nausea in Observation Group 1 (3.86 ± 2.2, 1.45 ± 1.21, and 2.45 ± 2.18) were lower than the other two groups (4.31 ± 1.58, 3.57 ± 2.00, and 3.70 ± 1.99; 4.59 ± 2.65, 4.51 ± 2.38, and 4.34 ± 2.32; *P* < 0.05). The VAS for shoulder pain in Observation Group 2 was lower than the Control Group (*P* < 0.05). Observation Group 1 showed a higher clinical recovery rate (72.15%) compared to the other two groups (43.66% and 31.58%, *P* < 0.05). The efficacy rate of the Observation Groups (87.34% and 88.73%) was considerably higher than the Control Group (69.74%, *P* < 0.05), while the difference between the two Observation Groups was insignificant (*P* > 0.05).

**Conclusions:**

The operation duration is the main factor that influences gastrointestinal dysfunction after surgery. Acupuncture can improve gastrointestinal function. It is advisable to initiate acupuncture as early as possible.

**Trial registration:**

N/A.

## Background

Postoperative gastrointestinal dysfunction (POGD), also identified as postoperative ileus (POI), is a rapid pathological alteration of the gastrointestinal tract resulting from laparoscopic surgical procedures. Clinically, it presents as symptoms such as nausea, vomiting, abdominal distension, delayed excretion or defecation, intestinal obstruction, gastrointestinal bleeding, and in severe cases, it may lead to intestinal infections and secondary multiple organ dysfunction [1].

Laparoscopy is widely utilized by surgeons in the surgical management of kidney diseases, for its advantages of minimal incision, reduced bleeding and speedy postoperative recuperation, which significantly ameliorate the efficiency and quality of treatment [2]. However, POGD due to intraoperative anesthetics and the establishment of artificial pneumoperitoneum [3], as well as the nervousness caused by surgery [4, 5], often hold back postoperative recovery. Studies have shown that 25% of patients after laparoscopic urinary surgery have postoperative abdominal distension [6], 80% of patients have shoulder pain [7], and the recovery time for eating and defecation without intervention after laparoscopic surgery is significantly delayed after laparoscopic surgery [3].

Traditional Chinese medicine (TCM) manipulations such as acupoint pressing [8], thumbtack needle, plaster application [9], and auricular acupuncture [10] are highly recommended by the ‘Expert Consensus on the Prevention and Treatment of Postoperative Gastrointestinal Dysfunction’ in 2021 [1] as they significantly accelerate postoperative recovery of intestinal peristalsis recovery and prevent postoperative abdominal distension. Clinically, we often use acupuncture to treat POGD. To further explore the acupuncture efficacy and related factors, we retrospectively analysed the clinical data of 226 inpatients who underwent laparoscopic nephrectomy at Qilu Hospital of Shandong University.

## Methods

### Study design and participants

This study was a retrospective analysis of medical records regarding gastrointestinal dysfunction after laparoscopic nephrectomy at Qilu Hospital, Shandong University, from January 1 to October 31, 2022. We had obtained approval from the hospital’s ethics committee for the collection of patient data (KYLL − 2022(ZM) − 699).

The medical records were sourced from the inpatient medical records of patients who underwent laparoscopic nephrectomy due to conditions such as renal tumors and kidney stones, with consistent pre - and post - operative diagnoses. POGD was diagnosed according to the diagnostic criteria established by Gomez - Izquierdo et al. in 2017 [11], which stated post - operative patients experience vomiting and/or abdominal distension, along with absence of exhaust, defecation, and/or inability to tolerate food intake.

The medical record materials were needed completely, including the admission record (recording information such as age, gender, disease course, underlying diseases, smoking and drinking history, etc.), the surgical informed consent form (recording the pre - operative diagnosis and the intended surgical method), the anesthesia record sheet (recording the anesthesia method, post - operative analgesia method, and intraoperative blood transfusion and fluid infusion), the surgical record sheet (recording the surgical method, operation duration, and surgeon’s name), the doctor’s order sheet (recording the patient’s medication, whether acupuncture was performed and the order time), the informed consent form for acupuncture treatment (recording the acupuncture time, acupoints selection, acupuncture angle and depth, needle - retaining time, number of acupuncture sessions, acupuncturist’s name, etc.), the nursing record sheet and the daily progress note (recording the time of the first post - operative exhaust, the occurrence and handling of post - operative reactions in each group) and the front page of the medical record (recording the postoperative diagnosis and pathological diagnosis).

Patients were excluded if they were not conformed to the diagnostic and inclusion criteria; suffered gastrointestinal diseases, such as gastrointestinal dysfunction, diarrhea, constipation, gastrointestinal paralysis, or had undergone gastrointestinal surgery before operation; with severe cardiovascular and cerebrovascular diseases, liver and kidney disfunction, diseases of endocrine, immune, and blood system, and advanced cancer cachexia; with wound infection, bleeding, gastrointestinal infection, bleeding, and secondary multiple organ dysfunction; used drugs or manipulations which had an impact on gastrointestinal function besides acupuncture perioperatively; or suffered mental illness.

In this study, 574 medical records of laparoscopic nephrectomy at Qilu Hospital of Shandong University from January 1 to October 31, 2022 were screened. Among them, 226 medical records met the above inclusion and exclusion criteria. The research - related valid information in the above - mentioned medical records (such as age, gender, disease course, operation duration, surgeon, anesthesia method, post - operative analgesia, blood transfusion status, underlying diseases, smoking and alcohol history, acupuncture timing, acupuncturist, etc.) was extracted and entered into the database. The finally selected valid cases all adopted the combined technique of endotracheal intubation general anesthesia and intravenous anesthesia. After the operation, a patient - controlled analgesia (PCA) pump was used. The surgery was performed by Surgeon X, and the acupuncture treatment was carried out by Acupuncturist Y.

### Categorization

According to the time when the acupuncture treatment orders were given in the doctor’s order sheet, all the medical records were divided into the acupuncture group (observation group) and the control group. Whether to issue a medical order for acupuncture treatment was determined by the surgeon according to patients’ physical condition.

### Interventions

According to the daily progress notes, doctor’s order sheets and nursing record sheets, patients in the Control Group were administered the ERAS protocol for urology, including preoperative fasting, intraoperative fluid management, multimodal pain management, and early mobilization.

Besides the ERAS protocol employed by the Control Group, the Observation Groups also used acupuncture at acupoints Zhongwan (CV 12), Neiguan (PC 6) and Zusanli (ST 36). Sterile acupuncture needles of φ 0.30 × 40 mm were used in all acupuncture treatment, and electronic acupuncture treatment instrument (SDV - IIB) manufactured by Suzhou medical appliance factory in China, was used for stimulation.

When needling, CV 12 was punctured 25–30 mm in depth perpendicularly, PC 6 on both sides at a depth of 10–15 mm perpendicularly, and ST 36 of both sides were needled to a depth of 25–30 mm perpendicularly. Electronic acupuncture treatment instrument was used to stimulate both ST 36 points with two outputs. The waveform was set continuous at a frequency of 1 Hz, and the intensity was adjusted based on the patient’s tolerance. The treatment lasted for 30 min.

The Observation Group was subcategorized into Early Acupuncture Group (Observation Group 1) and Delayed Acupuncture Group (Observation Group 2), based on the timing of the acupuncture intervention. Observation Group 1 pertains to patients received acupuncture within 24 h after surgery, while Observation Group 2 comprises those who commenced treatment within 24–48 h after surgery. When it came to deciding whether to carry out early or delayed acupuncture treatment, the medical team conducted a comprehensive assessment of the patient’s postoperative status.

### Outcomes

The primary outcome was the duration of the first postoperative anal exhaust and defecation, i.e., the time between the end of the surgery and the first anal exhaust and defecation. Secondly, Visual analogue scale (VAS) for the severity of symptoms, such as abdominal distension, shoulder pain, and nausea, etc. at the 72 - hour after surgery was compared. The score was 0–10 from low to high, and the higher the score was, the more obvious the symptoms were.

The symptomatic quantitative classification was developed following the ‘Guiding for Clinical Research of Chinese Medicine’ [12]. The severity of the symptoms was recorded 3, 2, 1 and 0 score respectively, which indicated severe, moderate, mild and asymptomatic. The details are listed in Table 1. Patients who had anal exhaust recovered, abdominal distension disappeared, taken in food without vomiting and abdominal distension, and voluntary defecation, were recognized cured. If the main indications disappeared completely, and the score reduced by over two - thirds, it was deemed markedly effective. If the main indications alleviated and the score reduced by over one - third, it was effective. If the indications did not improve significantly, or even worsened, it was deemed ineffective. The cure rate is the proportion of the number of cured and markedly effective patients in the total number of patients, and the total effective rate is the proportion of the number of cured, markedly effective, and effective patients in the total number of patients. The evaluation was conducted both prior to acupuncture and 72 h after surgery. For the Control Group, the pre-treatment evaluation was conducted within 6 h after surgery.

**Table 1:** Symptomatic quantitative classification

Symptoms	Mild	Moderate	Severe
Abdominal distension and pain	Intermittent mild distension or pain	Obvious swelling or pain but tolerable	Persistent unbearable pain which needs medication
Nausea and vomiting	Occasional nausea	Sometimes nausea, and occasional vomiting	Frequent nausea and sometimes vomiting
Belching and sour regurgitation	Occasionally	Sometimes	Frequently
Diet reduction	A quarter less	A third less	One half less
Fatigue and weakness	The body is slightly tired, and can insist on light physical activity.	Weak limbs, barely able to carry out daily activities.	The body is weak and unwilling to move all day long.
Chest distress and sigh	Slight chest distress, and occasional sighing.	Obvious chest distress, and sometimes sighing.	Stifling chest oppression, and frequent sighing.
Obstructed stool	A little	Obstructed	Apparently obstructed
Thin sloppy stool	Loose stool	Loose stool, 2–3 times a day	Loose stool, more than 4 times a day
Less and yellow urine	Yellow urine	Less and yellow urine	Dark yellow urine, and the volume is significantly reduced.

Notes: The severity of the symptoms was scored as followed: 0, asymptomatic; 1, mild; 2, moderate; 3, severe

Adverse events were detected via patients’ feedback and daily nursing care, and were documented in the medical record. This included the occurrence, procedure, and disappearance of adverse events, as well as the treating measures taken.

The above VAS score results are recorded in the nursing record sheet. The scores for symptom severity were assigned based on the content recorded in the nursing records.

### Statistical analysis

SPSS Statistics 26.0 software was used for analysis. The measurement data were expressed by mean ± standard deviation after normality test. One-way analysis of variance was used for comparison between groups after homogeneity test of variance. Welch test was used when variance was inconsistent. Pearson correlation analysis and correlation coefficient calculation method were used to analyze the effect of each factor on curative effect. Rank sum test was used to compare rank data. *P* < 0.05 was considered statistically significant.

## Results

### Analyses flow

226 medical records were collected from the urology ward of Qilu Hospital, Shandong University between January 1 and October 31, 2022. Besides 9 records were excluded according to the exclusion criteria. Each patient underwent laparoscopic nephrectomy surgery, and was subsequently sorted into Observation Group 1 (79 cases), Observation Group 2 (71 cases), and Control Group (76 cases), in accordance with physicians’ medical orders. The patients in the 3 groups had similar baseline characteristics (Table 2).

**Table 2:** Demographics and baseline characteristics

Factors			Group			*P* value
			Observation Group 1	Observation Group 2	Control Group	
Age (years)			57.17 ± 16.74	61.98 ± 12.85	52.56 ± 14.25	0.296
Gender		Male Female	48	44	43	0.596
			31	27	33	
Disease course (months)			4.75 ± 9.37	3.03 ± 6.02	3.31 ± 6.43	0.193
Operation duration (hours)			3.45 ± 1.55	3.10 ± 1.39	2.97 ± 1.16	0.290
Blood transfusion		Yes No	16	5	2	0.296
			63	66	74	
Underlaying diseases		Yes	59	53	64	0.312
		No	20	18	12	
Smoking history		Yes	35	36	37	0.695
		No	44	35	39	
Alcohol history		Yes	44	32	39	0.564
		No	35	39	37	

### Primary and secondary end points

The first anal exhaust time in Observation Group 1, Observation Group 2, and the Control Group was 40.59 ± 18.21 h (95% CI, 33.38 to 47.81), 54.54 ± 12.88 h (95% CI, 51.18 to 57.90), and 62.26 ± 15.79 h (95% CI, 58.55 to 65.97) respectively. The time for first defecation was 56.28 ± 12.21 h (95% CI, 49.73 to 62.82), 71.13 ± 11.29 h (95% CI, 68.24 to 74.02), and 78.36 ± 14.71 h (95% CI, 74.96 to 81.77) respectively for each group. Significant differences were observed in the time of first postoperative exhaust and defecation among the three groups (*P* < 0.001) (Table 3).

**Table 3:** Time of the first postoperative anal exhaust and defecation

Groups	Cases	First postoperative anal exhaust (hours)	95% CI		First defecation time (hours)	95% CI	
			Lower bound	Upper bound		Lower bound	Upper bound
Observation Group 1	79	40.59 ± 11.21	33.38	47.81	56.28 ± 12.21	49.73	62.82
Observation Group 2	71	54.54 ± 12.88	51.18	57.90	71.13 ± 11.29	68.24	74.02
Control Group	76	62.26 ± 15.79	58.55	65.97	78.36 ± 14.71	74.96	81.77
*P* value		0.000^*^			0.000^*^		

Notes: ^*^compared between every two groups, *P* < 0.05

The VAS scores for accompanying symptoms, such as abdominal distension, omalgia, and nausea, were significantly lower in Observation Group 1 as compared to Observation Group 2 and the Control Group 72 h after the surgery (*P* < 0.05). For omalgia of Observation Group 2, the VAS score was significantly lower than that of the Control Group (*P* < 0.05). And for abdominal distension and nausea, there was no significant difference between Observation Group 2 and the Control *g*roup (*P* > 0.05) (Table 4).

**Table 4:** Comparison of VAS of concomitant symptoms

Groups	Cases	abdominal distension	omalgia	nausea
Observation Group 1	79	3.86 ± 2.26^*△^	1.45 ± 1.21^*△^	2.45 ± 2.18^*△^
Observation Group 2	71	4.31 ± 1.58^△^	3.57 ± 2.00^*△^	3.70 ± 1.99^△^
Control group	76	4.59 ± 2.65	4.51 ± 2.38	4.34 ± 2.32

Notes: Compared with the Control Group, ^*^P < 0.05. Compared between Observation Group 1 and 2, ^△^*P* < 0.05

The recovery time of anal exhaust was measured in the Control Group using normal distribution analysis. The statistical analysis showed a significant effect of operating duration on the recovery time of anal exhaust (*P* < 0.05). Other factors like age, disease course, blood transfusion, underlying disease, smoking, and alcohol history did not have a significant impact on recovery time (*P* > 0.05). Acupuncture timing significantly affected the recovery time of anal exhaust (*P* < 0.05). No further statistically significant results were found for other factors, including age, disease course, blood transfusion, underlaying diseases, smoking and alcohol history, and postoperative abdominal distension (*P* > 0.05) (Table 5).

**Table 5:** Factors affecting recovery time of anal exhaust after operation

Factors		Group		*r* ^a^	*r* ^b^	*P* value ^c^	*P* value ^d^
		Observation Group	Control Group				
Age (years)		60.43 ± 14.30	52.56 ± 14.25	0.168	0.139	0.101	0.246
Disease course (months)		3.59 ± 7.26	3.31 ± 6.43	0.148	0.055	0.149	0.647
Operation duration (hours)		3.21 ± 1.44	2.97 ± 1.16	0.077	0.269	0.454	0.022^*^
Blood transfusion	Yes No	21	2	-0.082	-0.164	0.427	0.167
		129	74				
Underlaying diseases	Yes	112	64	-0.001	-0.157	0.989	0.187
	No	38	12				
Smoking history	Yes	56	37	-0.030	0.001	0.771	0.991
	No	94	39				
Alcohol history	Yes	57	39	-0.029	-0.119	0.779	0.321
	No	93	37				
Abdominal distension	Yes	126	N/A	0.007	N/A	0.946	N/A
	No	24					
Acupuncture timing	< 24 h	79		0.592		0.000^△^	
	< 48 h	71					

Notes: Abbreviations N/A: not applicable

^a^ correlation coefficient for Observation Groups

^b^ correlation coefficient for the Control Group

^c^*P* value for Observation Groups

^d^*P* value for the Control Group

^*^Operating duration had statistically significant on the recovery time of anal exhaust, *P* < 0.05

^△^Acupuncture timing had statistically significant on promoting anal exhaust after operation, *P* < 0.05

The recovery rate in Observation Group 1 surpassed that of Observation Group 2 and the Control Group (*P* < 0.05), while no significant differences were found between Observation Group 2 and the Control Group (*P* > 0.05). For the total effective rate, either Observation Group 1 or 2 had a higher rate than the Control Group (*P* < 0.05), and there was no significant statistical difference between the two Observation Groups (*P* > 0.05) (Table 6).

**Table 6:** Clinical efficacy evaluation of three groups

Groups	Cases	Cured	Markedly effective	Effective	Ineffective	Recovery Rate ( % )	Total Effective Rate ( % )
Observation Group 1	79	16	41	12	10	72.15^*△^	87.34^*^
Observation Group 2	71	3	28	32	8	43.66	88.73^*^
Control Group	76	6	18	29	23	31.58	69.74
*P*						0.000	0.002

Notes: compared with the control group, ^*^P < 0.05. Compared between observation group 1 and 2, ^△^*P* < 0.05

No patient in this study documented experienced needle fainting, stuck needles, broken needles, unbearable needling pain, infection, abscess, or any other needle-related adverse events.

## Discussion

The concept of ERAS was first proposed by Professor Henrik Kehlet of the University of Copenhagen in 1997. It involved implementing perioperative optimisation measures backed up by medical evidence, with the aim of reducing the physical and psychological trauma experienced by surgical patients and facilitating fast recovery. Although laparoscopic techniques had improved the quality of treatment, they had a direct impact on the postoperative recovery process due to the gastrointestinal dysfunction they caused. A study reported a persistent incidence of postoperative gastrointestinal dysfunction of around 20–30% [13].

Currently, the ERAS protocol during the perioperative period in the Department of Urology of our hospital includes preoperative fasting, intraoperative fluid management, multimodal pain management, and early mobilization, etc. The specific content is adjusted according to the different states of different patients during the perioperative period. The medical records included in this article should cover every content mentioned above.

Anal exhaust was an early sign of gastrointestinal function recovery. This study found that the time of first anal exhaust after laparoscopy without intervention was 62.26 ± 15.79 h, which accorded with similar reports [14, 15]. And statistically analysed, the operation duration was identified as the foremost factor in postoperative anal exhaust (*P* < 0.05), and it exhibited a positive correlated with the speed (*r* = 0.269). No further statistically significant effects on the recovery time of postoperative anal exhaust (*P* > 0.05) were observed for other factors, including age, disease course, blood transfusion, underlaying diseases, smoking and alcohol history, etc. In the future, we would increase the sample size and explore related factors more thoroughly.

Gastrointestinal dysfunction after laparoscopic nephrectomy can be characterised as ‘vomiting’, ‘intestinal paralysis’, ‘abdominal fullness’, ‘abdominal pain’, ‘regurgitation’ and ‘constipation’ in TCM, according to the symptoms, such as nausea, vomiting, abdominal pain, abdominal distension, constipation, etc. ST 36 and CV 12 were found the top two points on the frequency list [16]. ST 36 was the He - Sea of the Stomach Meridian and the Lower He - Sea point of the stomach. Experiments showed that electroacupuncture at ST 36 could effectively regulate microcirculation of stomach and duodenum [17].

CV 12 was the Front-Mu acupoint of the stomach and one of the eight confluent acupoints associating with the fu organs. Acupuncture at CV 12 might inhibit gastric acid secretion via the somatosympathetic pathway, and cause muscle relaxation via the somatosympathetic pathway [18]. Acupuncture of CV 12 plus ST 36 could ameliorate symptoms of delayed gastric emptying in diabetic gastroparesis (DGP) model rats, and regulate the expression of interstitial cells of Cajal (ICC) and the stem cell factor (SCF) in gastric antrum tissues [19]. Moxibustion at acupoints CV 12 and ST 36 could efficiently prevent indomethacin-induced gastric lesions in rats and this effect appeared to be dependent upon the integrity of C fibers [20].

PC 6 was the Luo-Connecting acupoint of the pericardium meridian, which could treat vomit and hiccup in clinic. Takahashi T reviewed that PC 6 might be beneficial to patients with functional dyspepsia (FD), whereas the antinociceptive effects of acupuncture at PC 6 and ST 36 might be beneficial to patients with visceral hypersensitivity [21]. The antiemetic effect of acupuncture at PC 6 was mediated via the central opioid pathway [22]. Acid secretion was reduced by acupuncture at PC 6 in patients with duodenal ulcer [23].

In this study, the first anal exhaust time in the Observation Groups were 40.59 ± 18.21 h (Observation Group 1), and 54.54 ± 12.88 h (Observation Group 2), while the first defecation time were 56.28 ± 12.21 h (Observation Group 1), and 71.13 ± 11.29 h (Observation Group 2), which were shorter than the time of the Control Group (*P* < 0.001). It suggested acupuncture can promote gastrointestinal peristalsis and accelerate postoperative anal excretion and defecation.

Modern medicine believed that the central and gastrointestinal inhibitory effects of CO_2_, surgical stress, narcotic, and many other factors are prone to cause gastrointestinal dysfunction, making patients’ postoperative gastrointestinal peristalsis slow. By enhancing the patients’ cellular immune system, it could promote the gastrointestinal function to recover soon [24]. Motilin had an important effect on gastrointestinal contraction, and acupuncture could up-regulate motilin level to regulate gastrointestinal peristaltic function [9]. Serum motilin content was negatively correlated with the duration of abdominal distension and anal exhaust time [25]. Timing of acupuncture was critical to efficacy [4]. This study showed that there existed statistically significant difference (*P* < 0.001) on acupuncture timing for anal exhaust between the two acupuncture groups. And timing was positively correlated with the rapidity of postoperative exhaust (*r* = 0.592). We should intervene acupuncture as early as possible after operation.

Whether to issue a medical order for acupuncture treatment was determined by the surgeon according to the patient’s physical condition. Factors such as the long operation duration, the complexity of the surgical procedure, and the involvement of the intestinal tract in the operation site, would prompt the surgeon to consider acupuncture treatment to help with postoperative recovery.

When it came to deciding whether to carry out early or delayed acupuncture treatment, the medical team conducted a comprehensive assessment of the patient’s postoperative status. This included evaluating the stability of vital signs, the degree of pain, the presence of any comorbid conditions, and the patient’s tolerance to invasive procedures. If the patient had only mild postoperative pain and there was no life - threatening problems immediately after the operation, the surgeon usually initiated acupuncture treatment in the early postoperative phase. This enabled the patient to start the recovery process at the earliest opportunity. If the patient experienced obvious discomfort after the operation, such as severe nausea and vomiting, or if there is instability in the cardiovascular system, the medical team may opt to postpone the acupuncture treatment. Generally, this is done after the patient’s condition has been stabilized, which is typically around 48 to 72 h after surgery. In some cases, a multidisciplinary consultation may even be required to handle the situation properly.

Acupuncture, as a supplement to the routine perioperative treatment protocol in urology, is considered effective. Through a retrospective analysis of medical records, we were able to determine the exact effectiveness and onset time of acupuncture. This positive analysis gave surgeons and acupuncturists confidence, and provided the motivation and foundation for further rigorous randomized controlled studies.

We conducted a retrospective analysis of the relevant information in the previous medical records so as to obtain authentic results. Since the number of surgeries we perform each year was substantial, after strict screening, we can still obtain a sufficient number of medical records that meet the inclusion and exclusion criteria, which enabled us to complete the preliminary clinical analysis. The retrospective analysis was just our starting point. In the future, we will concentrate on the clinical research of postoperative complications to make the research protocol more reasonable, rigorous, and explicit.

### Strengths and limitations

The strengths of this study lay in its controlled design and the medical records categorization, besides the context of perioperative period management. In the chosen records, the surgery and acupuncture were performed by the same surgeon and acupuncturist, to minimize heterogeneity among treatments of the same group. By retrospective analysis, we believe that the results added primary evidence supporting acupuncture effects in facilitating postoperative gastrointestinal function recovery. The study also had several limitations. Firstly, it was a retrospective analysis, and we would conduct a clinical trial with a multicenter, randomized controlled design, to further clarify the effect of acupuncture. Secondly, the existing definition of POGD was ambiguous, resulting in heterogeneity among studies and making accurate transverse comparisons difficult. Finally, the study lacked of potential factors that could affect the results and laboratory tests were not examined, and warrant further investigation.

9 medical records with complete documentation were excluded because they met the exclusion criteria. These cases accounted for 3% of the total number of cases initially included in the study. This proportion is relatively small, and the impact on the overall research results is within a controllable range. Moreover, all these excluded cases were from the control group. In this study, there were rarely excluded cases in the acupuncture group, which might be related to the high recognition and widespread application of acupuncture treatment in our hospital. This positive result also reflects the clinical guiding value of this article.

## Conclusions

In conclusion, this retrospective analysis demonstrated that electroacupuncture significantly shortened the time for first postoperative anal exhaust and defecation after laparoscopic nephrotomy. These findings confirmed and expanded previous evidence of the efficacy of electroacupuncture remained in the ERAS context. Electroacupuncture could be a potent complement to the ERAS protocol in enhancing recuperation after laparoscopic nephrectomy.

## Data Availability

The data and materials are available from the corresponding author ZHANG Xiang on reasonable request as a supplementary information file.

## Abbreviations

ERAS: Enhanced Recovery After Surgery
POGD: postoperative gastrointestinal dysfunction
EA: electroacupuncture
VAS: visual analogue scale
POI: postoperative ileus
TCM: Traditional Chinese Medicine
